# Map-Based Indoor Pedestrian Navigation Using an Auxiliary Particle Filter

**DOI:** 10.3390/mi8070225

**Published:** 2017-07-19

**Authors:** Chunyang Yu, Naser El-Sheimy, Haiyu Lan, Zhenbo Liu

**Affiliations:** 1 College of Automation, Haibin Engineering University, Harbin 150001, China; 2 Department of Geomatics Engineering, University of Calgary, Calgary, AB T2N 1N4, Canada; elsheimy@ucalgary.ca (N.E.-S); hlan@ucalgary.ca (H.L.); zhenbo.liu2@ucalgary.ca (Z.L.)

**Keywords:** map information, MEMS sensors, map aiding, map matching, auxiliary particle filter, cascade structure algorithm, indoor pedestrian navigation

## Abstract

In this research, a non-infrastructure-based and low-cost indoor navigation method is proposed through the integration of smartphone built-in microelectromechanical systems (MEMS) sensors and indoor map information using an auxiliary particle filter (APF). A cascade structure Kalman particle filter algorithm is designed to reduce the computational burden and improve the estimation speed of the APF by decreasing its update frequency and the number of particles used in this research. In the lower filter (Kalman filter), zero velocity update and non-holonomic constraints are used to correct the error of the inertial navigation-derived solutions. The innovation of the design lies in the combination of upper filter (particle filter) map-matching and map-aiding methods to further constrain the navigation solutions. This proposed navigation method simplifies indoor positioning and makes it accessible to individual and group users, while guaranteeing the system’s accuracy. The availability and accuracy of the proposed algorithm are tested and validated through experiments in various practical scenarios.

## 1. Introduction

Reliable indoor pedestrian navigation systems require devices and algorithms that can provide accurate, continuous, autonomous and stable position solutions in indoor environments. The indoor navigation system can effectively decrease the time and energy consumed to maneuver through large indoor environments, such as airports, hospitals, malls, and museums [1,2]. Moreover, precise indoor location results can help emergency workers perform urgent tasks by reducing the amount of time it takes to navigate these environments. Police, firefighters, and first responders could benefit from applications that provide navigation solutions for indoor search and rescue. In addition to how accurate positioning can satisfy a user’s basic needs, indoor navigation can also be connected with customer relationship management (CRM) systems to explore the deep and added value of big data [3]. Therefore, the market demand for indoor navigation systems is large and still growing, and more industrial companies, institutions, and universities are now focusing their research efforts on indoor pedestrian navigation [4,5,6,7,8].

Currently, the normal techniques for indoor navigation are radio frequency (RF)-based techniques and inertial navigation techniques. RF-based systems include Wi-Fi, Bluetooth, and radio-frequency identification (RFID)-based methods [9,10]. The Wi-Fi-based technique appears to be the most popular of all RF-based methods because of its ubiquitous presence in smartphones and extensive coverage in modern cities [11,12]. RF-based systems are not self-contained systems; they need aided infrastructures, which will cost a considerable amount of time and money to install, maintain, pre-survey and update [10]. Moreover, most of the infrastructure that RF-techniques rely on need electricity power to run.

Navigation using inertial sensors is a totally self-contained navigation technique, as it is not susceptible to external environments and does not need any infrastructure [13,14]. Moreover, microelectromechanical systems (MEMS)-based inertial measurement unit (IMU) data has already been built into many smartphones [15]. These sensors have the benefits of low cost, miniaturization, and low power consumption [16,17]. Due to the relatively low accuracy of MEMS sensors and the principle of inertial navigation, the navigation errors of MEMS-based IMUs grow significantly with time. If the unique human kinematic walking model is considered, zero velocity update (ZUPT) and non-holonomic constraints (NHC) can be used to calibrate the sensor drift error [18]. However, the heading drift error of a standalone inertial navigation system, which may cause a serious problem in the final navigation solution, still cannot be well estimated. Therefore, inertial navigation systems should be integrated with other sensors or aiding information [19,20].

To overcome the drawbacks of standalone inertial navigation systems, a series of aiding information, such as cellular networks (2G/3G/4G), a IEEE 802.11-based Wi-Fi network, Bluetooth, and iBeacon, are widely integrated with MEMS-based inertial sensors [21]. A number of such systems can now be seen in various industrial products such as Indoo.rs^TM^, Infsoft^TM^, Eyedog^TM^, Meridian^TM^, and Pole Star^TM^. However, the main drawback of these methods is the obvious requirement of building dependent infrastructures and power. For example, iBeacon, and Bluetooth-based methods are mostly restricted by infrastructure installation and maintenance [22], and they cannot work in places without signal coverage. Moreover, Wi-Fi- and magnetic-based methods may require a considerable amount of time to survey a structure, and build and update the database [23]; therefore, these methods are not considered in this paper.

One of the most widely available and commercial means of navigation is using maps that need no additional infrastructure or equipment. Currently, most of public buildings, such as airports, hospitals and universities, offer digital maps. The customers can utilize the corresponding quick response code (QR code) to download the building map information. Meanwhile, nowadays, Google Map^TM^, Openstreet Map^TM^, and many other location service companies can provide open-source indoor maps [24]. Map information is the crucial prerequisite for indoor map-aided navigation. Even when the map information source is inaccessible, the user can take a picture of the floor plan map, and easily get the digital map information through image processing technique (e.g., edge detection and Hough transform) [25].

To date, for most of indoor navigation products, map information is not involved in position calculation, but rather is used to plot the position solution or the destination in the “presentation layer” of the basic algorithm. However, maps can also be further used in navigation algorithms through data fusion techniques [26,27]. Furthermore, indoor maps can act as constraints that correct navigation errors. In outdoor environments, map information usually works as binary/geometric constraints to a preliminary navigation solution [28,29]. Generally, there are two commonly used methods to combine map information with the INS solutions: map-matching (MM) and map-aiding (MA) methods. MM is widely used for fixed trajectory applications such as vehicle navigation in outdoor environments [30,31,32], which assumes that the object moves within a known and restrictive path. Compared with MM, MA is more flexible for indoor navigation and does not require any assumptions about the trajectory, but it is not as powerful as MM [33]. Therefore, in this research, both methods are used to complement each other. 

Map-aided navigation has attracted a great deal of interest for indoor navigation. Amrit Bandyopadhyay et al. use a comprehensive database of building features and landmarks, which might contain floor plans and the features in the building such as hallways, stairwells, elevators, etc., to discontinuously increase the position accuracy [34]. Li Tao et al. use map information from a suitable source, and the internal map data structure may include the map projection parameters, geometric shapes, identifications (IDs) and information type of all the entities in the map [35]. The indoor map information we use is different from these methods, and simply represented by a set of line segments, which makes the map easy to access and convenient to use. A simple map can also avoid adding computational burden or complexity to the system.

The common solution for integrating INS with aiding information is to use the Kalman filter (KF), specifically the Extended Kalman filter (EKF) [36,37]. Compared with the other filters, such as the unscented Kalman filter, or the particle filter (PF), KF is relatively less complex mathematically and easier to implement. However, KF has limited capabilities in providing accurate positioning on nonlinear integrated navigation systems. To enhance the performance of INS/map-integration systems, nonlinear estimation techniques that do not require linearized dynamic models should be considered. PF is a completely nonlinear state estimator, which can deal with nonlinear non-Gaussian models [34]. PF utilizes the Monte Carlo approach to approximately model the probability density distribution through a large number of samples. Therefore, it can accommodate arbitrary sensor characteristics, motion dynamics, and noise distributions, and avoid the linearization done in EKF. Moreover, PF can accommodate large orientation uncertainty [35], which is typical when dealing with low-cost gyros. More importantly, PF is flexible in exploiting aided indoor map information to the INS. These advantages motivate the use of PF for the INS/map-integration system. On the other hand, PF suffers from computational burden issues and sample impoverishment problems [38]. Thus, while both KF and PF have their weaknesses and strengths, incorporating them together can overcome their shortages and build on their advantages [39].

Given this background, this research proposes a low-cost and continuous indoor navigation using both MM and MA as aiding information for MEMS-based inertial navigation systems. To the best of our knowledge, there have been few reports about using MM and MA methods cooperatively [40]. Moreover, there is no real product in the market that uses only MEMS inertial sensors and map information to realize indoor navigation. Furthermore, in this research, taking the merits of KF and PF into consideration, both KF and PF will be used in different stages of the pedestrian navigation system through using a cascade structure algorithm.

In this paper, a totally self-contained and low cost indoor navigation system is proposed. Through integrating indoor map information and built-in smart-phone IMU, we present a self-dependent and easy to implement indoor-positioning method. This technology has direct application for the everyday user, ranging from indoor shopping to the navigation of large buildings like airports and museums, and could assist emergency rescuers. Currently, for building owners who want to provide indoor navigation for customers, the cost of these systems is high because they require technicians to install and maintain infrastructure. In addition, these systems are affected by electronic interference and require pre-survey data that degrades over time and needs to be constantly updated. However, smartphones equipped with MEMS sensors and map information require no infrastructure and are self-contained. This resolves current issues with indoor navigation systems, and creates an easier and more economical way for business owners to implement them. 

To be more specific, the contributions of the paper are:

- Firstly, only smartphone built-in IMU and indoor map information are used in the proposed algorithm, and no pre-surveying or structure installation are needed for this system, which can dramatically reduce the time and economic cost of indoor navigation systems;
- Secondly, the KF and PF are combined to effectively utilize MEMS sensor data. A cascade structure algorithm is proposed to decrease the number of the particles in the PF, which can indirectly decrease the system computational burden;
- Lastly, the map-matching and map-aiding methods are innovatively combined, and the combination method can make full use of indoor map information which, to a certain extent, will improve the navigation calculating precision;
- At the end of the work, experimental results in different scenarios are used to show the benefits of the proposed algorithm.

The organization of this paper is as follows: a brief description of the cascade structure algorithm is provided in Section 2. The lower layer KF and upper layer PF are then respectively discussed in this section, too. Followed by the MM and MA methods on the upper layer. In Section 3, the real experiments are performed to test the proposed methods. In Section 4 and Section 5, the performance of the original approach is analyzed through field experiments, and the conclusions are given.

## 2. Algorithm

### 2.1. Cascade Structure Algorithm

**Figure 1:**
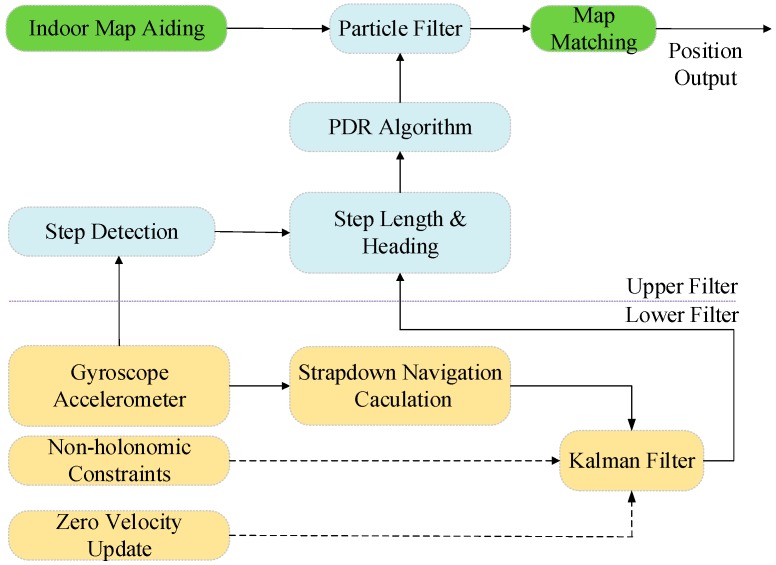
Cascade structure of map/INS integration algorithm using the Kalman filter (KF) and the particle filter (PF).

This research uses indoor maps integrated with built-in MEMS sensors in smartphones by using an auxiliary particle filter (APF). To take advantage of the merits of the KF and overcome the implementation issues of the PF, such as computational burden and particle impoverishment, a cascade-connected KF and APF integration algorithm comprised of a two-layer architecture is proposed in this research, as shown in Figure 1 [41,42].

By changing the rate operation of the upper PF, the computational burden of the PF will be greatly reduced; therefore, the time it costs to estimate the navigation solution will be reduced. However, to take advantage of high-frequency sensor data, the lower KF will perform an update cycle according to the high rate inertial sensors measurements. Moreover, the underlying KF uses ZUPT and NHC as inputs to correct the preliminary INS navigation solution [2,43]; therefore, a better INS solution can be provided to the upper particle filter. The upper PF applies the pedestrian dead-reckoning method to calculate the pedestrian’s position [44]. The step-length change and heading change, which are calculated using navigation results in the underlying filter, are used to update the nonlinear state model of the upper layer’s filter. A priori known map information is considered as an independent measurement to correct the lower filter solutions, and the relationship between two layer filters is shown in Figure 1. In the lower KF, we select the pedestrian’s three-dimensional position, velocity and attitude information as the state vector; for the upper APF, taking the computational burden into consideration, there is only two-dimensional position information in the state vector.

### 2.2. Lower Kalman Filter

In the lower filter, the strapdown inertial navigation method is used to solve the preliminary indoor position. Then, ZUPT and NHC can be used to correct these initial results through the KF according to the user’s motion state. The NHC technique is used to constrain the lateral and vertical velocities of the moving pedestrian. It is assumed that lateral and vertical speeds are zero, based on the fact that a moving platform cannot skid or jump. Therefore, the forward, lateral, and vertical speeds work as the velocity update for the INS to limit the velocity errors. Moreover, if the pedestrian is in static mode and is detected correctly, the ZUPT technique will be triggered and applied for INS error correction. For a more detailed analysis of the use of ZUPT and NHC, refer to [2,7]. The discrete measurement model of the lower KF is:

(1) $$\delta z_{k}^{ins/zupt}=H_{k}^{ins/zupt}\delta x_{k}^{ins}+v_{k}^{ins/zupt}$$

where $\delta z_{k}^{ins/zupt}$ is the measurement vector, which is the true velocity of the system minus the velocity vector derived from INS during the system’s static mode, $\delta x_{k}^{ins}$ is the state of the system, which contains the position, velocity and attitude, and $v_{k}^{ins/zupt}$ is the measurement noise matrix. The measurement matrix $H_{k}^{ins/zupt}$ is:

(2) $$H_{k}^{ins/zupt}=\left[\begin{array}{ccc} 0_{3\times 3} & I_{3\times 3} & 0_{3\times 3} \end{array}\right]$$

every time the state error $\delta x_{k}^{ins}$ is updated, it is applied to the INS navigation solution, and $\delta x_{k}^{ins}$ is reset. The upper filter’s step detection is performed during the INS mechanization process, using the method in [45]. The stride length is computed using the navigation solution provided by the lower KF. Specifically, the distance between every two coordinates when the pedestrian is static is defined as the stride length:

(3) $$S_{k}=\sqrt{{\left(x_{step\_m}-x_{step\_m-1}\right)}^{2}+{\left(y_{step\_m}-y_{step\_m-1}\right)}^{2}}$$

in which $\left(x_{step\_m},\;y_{step\_m}\right)$ is the position estimated from the lower KF. The heading change of each stride is the difference between every two heading solutions provided by the KF when the pedestrian is static. It employs the equation:

(4) $$\delta \psi_{k}=\psi_{k}-\psi_{k-1}$$

therefore, the measurement model of the PF can be expressed as:

(5) $$\{\begin{array}{c} {\hat{S}}_{k}=S_{k}+v_{Sk}\\ \delta {\hat{\psi }}_{k}=\delta \psi_{k}+v_{\psi k} \end{array}$$

in which $v_{Sk}$ and $v_{\psi k}$ are the noise of the stride length and heading change independently. That is, the stride length and the heading error both have a corresponding error model describing the uncertainty of the PF observation. The model is specified by distribution $v_{S}$ of the error $v_{Sk}$ and $v_{\psi }$ of the error $v_{\psi k}$. To simplify the question, we assume that $v_{Sk}$ and $v_{\psi k}$ are both normally distributed. According to [46], the standard deviations are closely related to the heading change.

### 2.3. Upper Particle Filter

**Figure 2:**
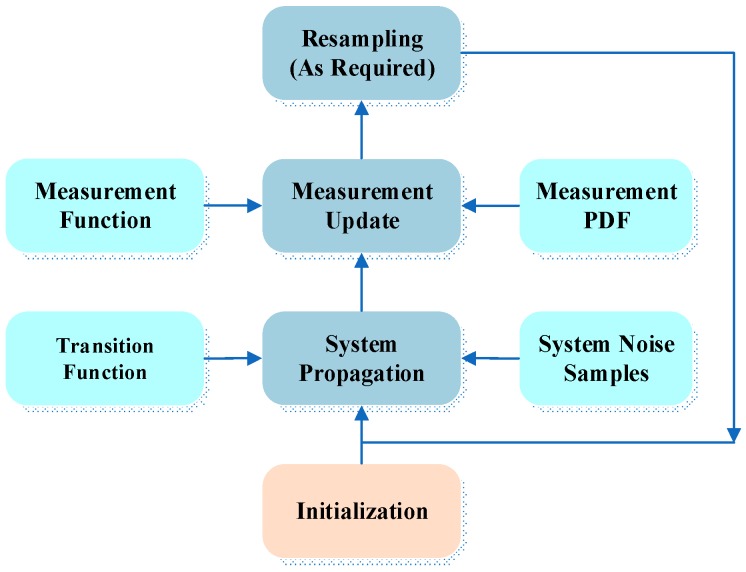
Three phases of the particle filter.

The PF was invented to numerically implement the Bayesian estimator. As shown in Figure 2, there are three phases in the PF: system propagation, measurement update and resampling (if needed) [47,48]. Rather than applying prior probabilities in Bayes estimation, it employs a set of particles with values and weights to approximately represent $p(x_{k}|Y_{k})$ through the Monte Carlo sampling approach. This allows for the PF maps’ intractable integral Bayesian solution to tractable discrete sums of weighted samples drawn from the posterior distribution. The posterior density function (PDF) $p(x_{0:k}|Y_{k})$ of the estimated state can be represented by:

(6) $$p(x_{0:k}|Y_{k})\approx \overset{N}{\underset{i=1}{\sum }}w_{k}^{i}\delta \left(x_{0:k}-x_{0:k}^{i}\right)$$

where parameter N is the number of particles, and it is chosen by the user as a trade-off between computational effort and estimation accuracy. $Y_{k}$ is the set of the system measurement $y_{k}$ at time *k*. $w_{k}^{i}$ is the weight of the relative particle, and $\sum_{i=1}^{N}w_{k}^{i}=1$. The approximate representation of $w_{k}^{i}$ is:

(7) $$w_{k}^{i}\propto w_{k-1}^{i}\frac{p(y_{k}|x_{k}^{i})p(x_{k}^{i}|x_{k-1}^{i})}{q(x_{k}^{i}|x_{k-1,}^{i}y_{k})}$$

where $q\left(x_{k}^{i}|x_{k-1}^{i},y_{k}\right)$ is the importance density function (IDF), a standard particle filter selecting priori density function as the importance density function, simplifying $w_{k}^{i}$ as:

(8) $$w_{k}^{i}\propto w_{k-1}^{i}p(y_{k}|x_{k}^{i})$$

However, as the new measurement is not taken into consideration in the IDF, the samples from the IDF and the samples from the real PDF have a significant difference, which will cause a sample impoverishment problem. Although resampling can alleviate this problem, it can also increase the system’s computational burden.

In this research, the APF is used in the upper filter. Different from the traditional PF algorithm, the APF takes the importance distribution of the current measurements into consideration. The APF can provide an efficient way to solve the particle impoverishment problem through introducing an IDF $q\left(x_{k},i{|Y}_{k}\right)$, which can be represented as [48]:

(9) $$q\left(x_{k},i{|Y}_{k}\right)\propto p(Y_{k}|\mu_{k}^{i})p\left(x_{k}|x_{k-1}^{i},y_{k}\right)w_{k-1}^{i}$$

The weight of the particle can be calculated from Equation (10):

(10) $$w_{k}^{i}\propto \frac{\left(Y_{k}^{i}|x_{k}^{i}\right)}{p(Y_{k}|\mu_{k}^{ij})}$$

where $\mu_{k}^{i}$ is the statistical characterization of $x_{k}$ based on $x_{k-1}^{i}$ [47]. This IDF promotes diversity in the population of particles. Compared with standard PF, APF performs weighting manipulation twice, and the weights of the APF particles are more stable than that of the standard PF. Therefore, APF estimated solutions are more accurate than PF in this application. 

In this research, the state model we use for APF is the dead reckoning (DR) positioning update model. The pedestrian dead reckoning (PDR) is implemented through exploiting the kinematic features of a pedestrian’s gait with the traveled distance and heading information. Essentially, the PDR is the determination of a new position using the knowledge of a previously known position together with the current traveled distance and heading information. The PDR propagation equation is:

(11) $$\{\begin{array}{c} E_{k+1}^{i}=E_{k}^{i}+S_{k}^{i}\sin\left(\psi_{k}^{i}\right)\\ N_{k+1}^{i}=N_{k}^{i}+N_{k}^{i}\cos\left(\psi_{k}^{i}\right) \end{array}$$

In Equation (11), the stride length $S_{k}^{i}$ and heading $\psi_{k}^{i}$ calculated from the lower filter are used to transfer and update particles from time k to time k+1. After the system propagation, the weights of particles are updated during measurement update process through using the MA method, which will be explained in the next section.

### 2.4. Map-Matching and Map-Aiding Methods

In this paper, MA and MM methods are innovatively combined to enhance the accuracy of the final position solution by making full use of the indoor map information. MA utilizes indoor map information as a measurement resource during the measurement update process to amend the weight of particles in the APF. MM uses map information to decrease the predicted position errors by matching the estimated solution to the existing map [26,49,50].

#### 2.4.1. Map-Aiding Implementation

For indoor pedestrian navigation implementation, indoor architectural plane information could be used to constrain a pedestrian’s trajectories, thus decreasing the uncertainty and improving the accuracy of the final navigation solutions by updating the weight of the particles. In the APF, the relative weight of each particle, $w_{k}^{\left(i\right)}$, is updated by comparing the predicted measurements with the probability distribution function obtained from the actual measurement process. Similarly, in this research, the weight $w_{k}^{\left(i\right)}$ of a propagated particle is enforced by indoor map information constraints. A cross-wall method based on indoor map matching is used [51]. The “cross-wall” problem is solved based on the development of a floor map-aided APF algorithm by weighing the particles. After system propagation, if the line segment between the new generated particle and the previous one intersects with the wall boundary, then this particle is invalid, and its weight will be assigned as zero:

(12) $$w_{k}^{\left(i\right)}=0$$

If a wall does not intersect with the particle during the propagation step, this means the pedestrian navigation system user is still in the previous room/office/corridor. In this case, the particle’s weight remains the same with the weight of the previous step. It is important to mention that the cross-wall detection method is of crucial importance for the whole MA method, as incorrect detection will introduce wrong particles into the next step. This step is repeated for all particles.

To test if the new particle passes the wall of the building or not, an effective particle detection algorithm is designed. In this research, the map data was provided by the University of Calgary (U of C). The data provided is in shape files format and consists of latitude and longitude coordinates. This data shape can display geographic information (including point, line, face, polygons, polyhedron and model) and consists of 3D geographic coordinates: longitude, latitude and altitude. By using the Mapping Toolbox of MATLAB 2013, the map data in the shape file could be easily loaded and converted into x and y coordinates in meters, and these coordinates can be saved as vectors in matrices. In this way, the effective particle detection problem can be changed into a relation analysis problem between particles and map information vectors. Firstly, let $A=\left(x_{k-1}^{\left(i\right)},y_{k-1}^{\left(i\right)}\right)$ and $B=\left(x_{k}^{\left(i\right)},y_{k}^{\left(i\right)}\right)$ separately represent coordinates derived from particle i at step k−1 and step k. Point A and B are assumed to be the start and end points of the step vector AB in the horizontal plane. Meanwhile, point A is assumed to be estimated from an effective particle, that is to say, point A is a reasonable position. 

**Figure 3:**
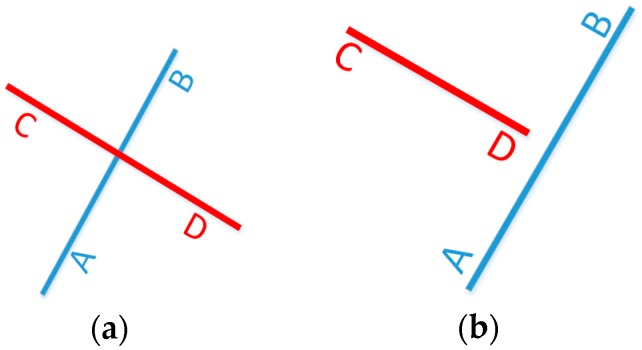
Two intersectant line segments (**a**) and two disjoint line segments (**b**).

As shown in Figure 3a,b, vector CD is one line segment of the map data. If the vector AB and CD intersect and meet one of the following conditions, then we can say that this new particle is not effective.

**Figure 4:**
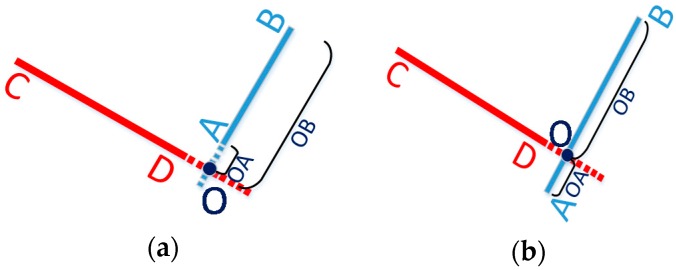
Examples of two intersectant line segments (**a**) and two disjoint line segments (**b**).

1. Point A and B should be on different sides of the line segment vector CD; Point C and D should be on different sides of the line segment vector AB. To judge whether one point is in the right or left side of a line segment, the following equations are used:
  - For vector $\left(x_{1},y_{1}\right)\to \left(x_{2},y_{2}\right)$, M is defined as $M=x_{1}\left(y_{3}-y_{2}\right)+x_{2}\left(y_{1}-y_{3}\right)+x_{3}\left(y_{2}-y_{1}\right)$.
  - If M=0, point $\left(x_{3},y_{3}\right)$ is on the line segment $\left(x_{1},y_{1}\right)\to \left(x_{2},y_{2}\right)$;
  - If M>0, point $\left(x_{3},y_{3}\right)$ is on the right side of the line segment;
  - Otherwise, point $\left(x_{3},y_{3}\right)$ is on the left side of the line segment.
2. Line AB and line CD should have one point of intersection. As shown in Figure 4a,b, O is the intersection of vector AB and CD, and OA, OB, and AB separately represent the length of the corresponding line segment vector. If AB > max{OA,OB} and CD > max{OC,OD}, O denotes an intersection point of line segments AB and CD.

#### 2.4.2. Map-Matching Implementation

MM is the process of utilizing a digital road network map database to improve the predicted position errors during the integration process by projecting the estimated positon to the priori digital map. In this research, a probability pedestrian trajectory map consisting of line segments is built from the indoor map. When the distance between the user’s current estimated position and the map line segment is within a certain threshold, then the MM method will be used to project the estimated position to the relative segment link. The threshold is determined by empirical value. If there is more than one map line segment satisfying that the distance between them and the estimated position are within the threshold, then in this epoch, the system will not perform the MM method.

There are three main techniques for MM. These are point-to-point matching, point-to-curve matching, and curve-to-curve matching. Which one to use is heavily dependent on how the data or network is structured. Taking the format available in this research (line segment) into consideration, the point-to-point matching algorithm is used in this research. The algorithm projects the estimated location, P, to the closest link in the network by using the following distance equation [41]:

(13) $$Distance=\frac{\left|x_{e}\left(y_{s}-y_{d}\right)-y_{e}\left(x_{s}-x_{d}\right)+\left(x_{s}y_{d}-x_{d}y_{s}\right)\right|}{\sqrt{{\left(x_{s}-x_{d}\right)}^{2}+{\left(y_{s}-y_{d}\right)}^{2}}}$$

(14) $$x_{P}=\frac{x_{e}\left(x_{d}-x_{s}\right)-y_{e}\left(y_{s}-y_{d}\right)-\left(y_{s}-y_{d}\right)\left(x_{s}y_{d}-x_{d}y_{s}\right)}{\left[{\left(x_{s}-x_{d}\right)}^{2}+{\left(y_{s}-y_{d}\right)}^{2}\right]{\left(\left(x_{d}-x_{s}\right)\right)}^{-1}}$$

(15) $$y_{P}=\frac{x_{e}\left(x_{d}-x_{s}\right)-y_{e}\left(y_{s}-y_{d}\right)-\left(x_{s}-x_{d}\right)\left(x_{s}y_{d}-x_{d}y_{s}\right)}{\left[{\left(x_{s}-x_{d}\right)}^{2}+{\left(y_{s}-y_{d}\right)}^{2}\right]{\left(\left(y_{d}-y_{s}\right)\right)}^{-1}}$$

in which $\left(x_{e},y_{e}\right)$ is the position estimated from the MA-based APF algorithm, and $\left(x_{s},y_{s}\right)$ and $\left(x_{d},y_{d}\right)$ are the start point and end point of a line segment in the digital map. Equation (13) is used to calculate the distance between the estimation position and the previously known line segment, and for detecting the line segment on which the estimated positon should be projected; Equations (14) and (15) are used to project coordinates to obtain the foot of a perpendicular, namely, a projection point.

## 3. Experiment and Results

The validity and feasibility of the proposed algorithm are tested by conducting ground experiments using different smartphones. Multiple scenarios were used to investigate the algorithm’s reliability and accuracy. The first test took place on the third floor of the Calgary Centre for Innovative Technology (CCIT) building at the University of Calgary, and the second and third tests took place on the first floor of the Energy Environment Experiential Learning (EEEL) building at the University of Calgary. The proposed algorithm has been implemented on the Samsung Galaxy Note 4 and Xiaomi smartphones. The used sampling rate of the IMU in the tests is 40 Hz. The devices’ parameters are shown in Table 1. In addition, to evaluate the accuracy of the final solution and quantitatively show the performance of the proposed algorithm, a reference trajectory generated method proposed in [2] is used in this research, which is based on the accelerometers and gyroscopes. 

**Figure 5:**
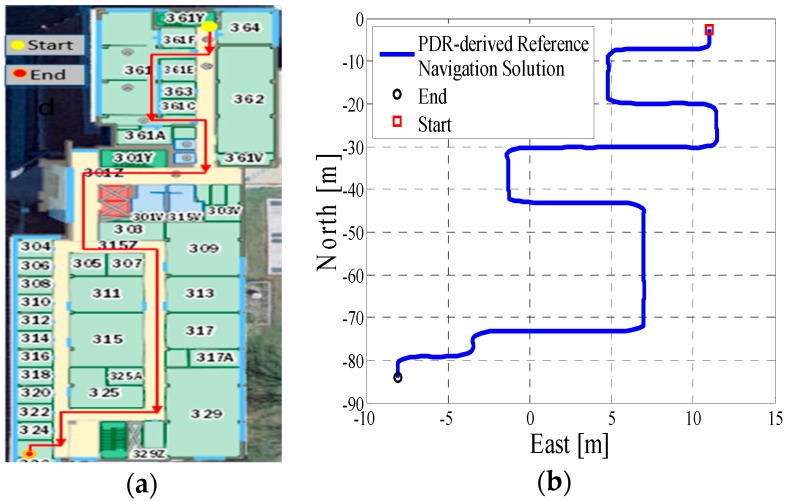
(**a**) Designed test trajectory on the third floor of the Calgary Centre for Innovative Technology (CCIT) building; (**b**) Reference trajectory for the test trajectory on the third floor of the CCIT building.

**Figure 6:**
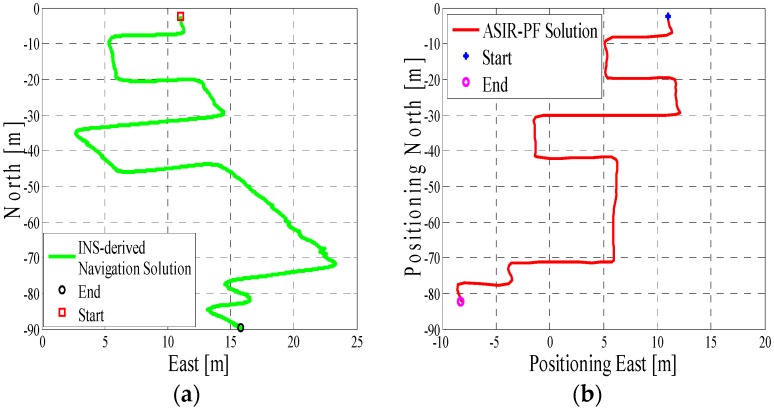
(**a**) Lower filter INS-derived navigation solution in the CCIT building; (**b**) The upper filter map-aiding (MA) method-derived position for design trajectory in the CCIT building.

**Figure 7:**
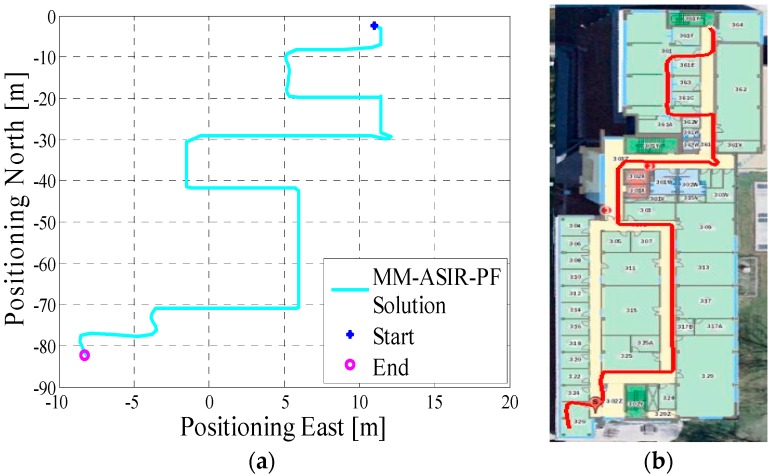
(**a**) MA–map-matching (MM)-derived navigation solution for test 1 in the CCIT building; (**b**) The final estimated position of test 1 in the CCIT building plotted on the digital map.

In test 1, the Xiaomi 3 smartphone is used to collect the pedestrian’s motion data. We carried the smartphone by hand. We used the hand-held motion, in which the smartphone was kept almost level in front of the user’s chest. A manual mode is used to provide the initial position information, which means that the user can manually enter the initial position and head according to the given map information. The experiment’s total walking distance is 120 m. Figure 5a shows the designed test trajectory on the third floor of the CCIT building, and Figure 5b is the corresponding reference trajectory derived using the method described in [2]. Figure 6a,b, respectively, are the lower filter-derived position and the proposed MA-only method derived position for test 1 in the CCIT building, and the number of particles used in this test is 1000. In addition, Figure 7 depicts the MA–MM-derived navigation solution for test 1, and the same position solution plotted on the digital map.

**Figure 8:**
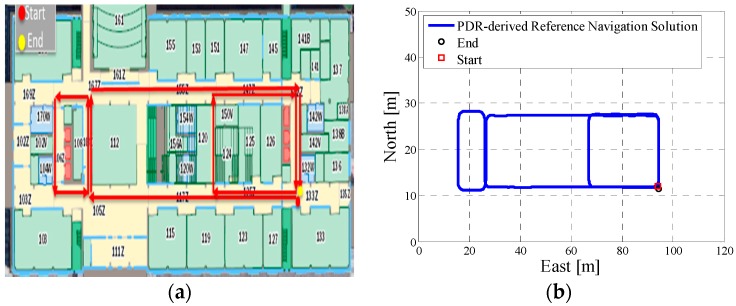
(**a**) Designed test trajectory on the third floor of the CCIT building; (**b**) Reference trajectory for the test trajectory on the third floor of the CCIT building.

In test 2, the Xiaomi 3 smartphone is used to collect the pedestrian’s motion data. The experiment’s total walking distance was 290 m. Figure 8a shows the designed test trajectory on the first floor of EEEL building, and Figure 8b is the corresponding reference trajectory.

**Figure 9:**
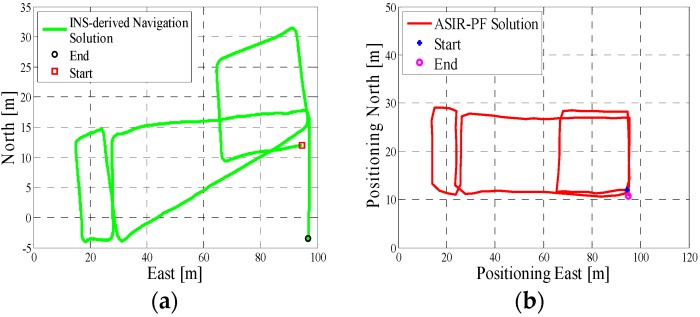
(**a**) Lower filter INS-derived navigation solution for test 2 in the Energy Environment Experiential Learning (EEEL) building; (**b**) The upper filter MA method-derived position for test 2 in the EEEL building.

**Figure 10:**
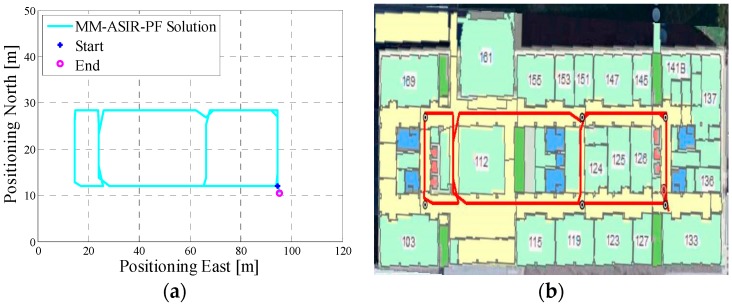
(**a**) MA–MM derived navigation solution for test 2 in the EEEL building; (**b**) The final estimated position of test 2 in the EEEL building plotted on the digital map.

Figure 9a is the lower filter INS-derived navigation solution for test 2 in the EEEL building, and Figure 9b is the proposed MA-only method derived position for test 2, and the number of particles used in this test is 1000. Figure 10 depicts the MA–MM derived navigation solution for test 2, and the same position solution plotted on the digital map. 

**Figure 11:**
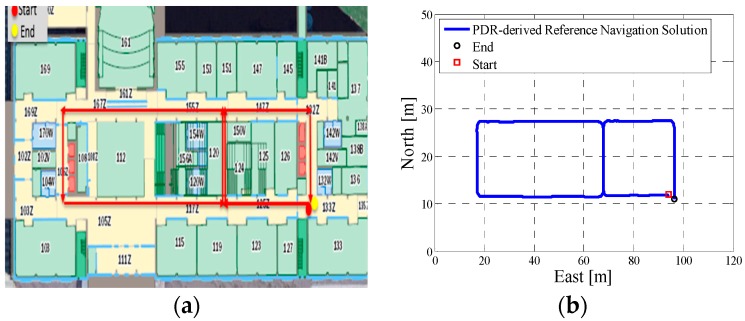
(**a**) Designed test trajectory for test 3 in the first floor of the EEEL building; (**b**) Reference trajectory for the test trajectory on the third floor of the CCIT building.

**Figure 12:**
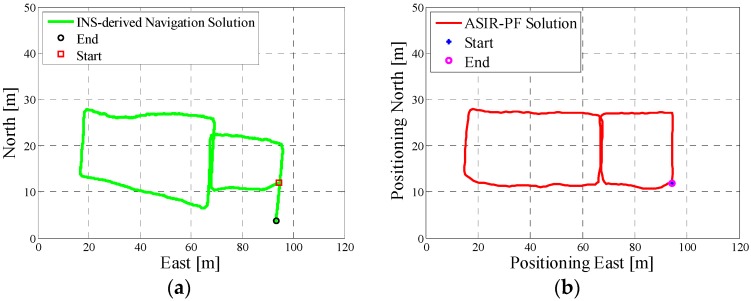
(**a**) Lower filter INS-derived navigation solution for test 3 in the EEEL building; (**b**) The upper filter MA method derived position for test 3 in the EEEL building.

**Figure 13:**
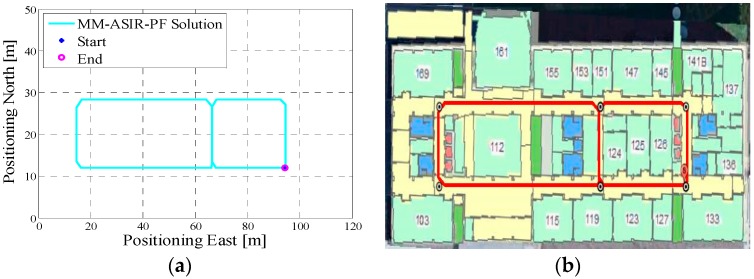
(**a**) MA-MM derived navigation solution for test 3 in the EEEL building; (**b**) The final estimated position of test 3 in the EEEL building plotted on the digital map.

In test 3, the Samsung smartphone is used to collect the pedestrian’s motion data. The experiment’s total walking distance is 210 m. Figure 11a shows the designed test trajectory on the first floor of the EEEL building, which is different from test 2, and Figure 11b is the corresponding reference trajectory. Figure 12a is the lower filter INS-derived navigation solution for test 2 in the EEEL building, and Figure 12b is the proposed MA-only method derived position for test 2, and 1000 particles are used to obtain this solution. Figure 13 are the MA–MM derived navigation solution for test 3 and the same position solution plotted on the digital map.

To quantitatively analyze the influence of the MM method on the final solution, different number of particles (N = 1500, 800, 600, 500) are used in the program for test 3 to verify its impact on the positioning errors. Table 2 gives the performance of the MM-only and MM–MA estimation solution.

## 4. Discussion

In the experiment section, different smartphones were used to collect the IMU data, and the presented algorithm has no special requirements for the MEMS sensors. If we compare the INS-derived solution with the designed test trajectory, (i.e., comparing Figure 5a with Figure 6a, Figure 8a with Figure 9a, Figure 11a with Figure 12a), it is obvious that the lower filter cannot provide a satisfying estimated position. Because of the standalone low-cost MEMS inertial navigation system, when ZUPT or NHC is used to correct the system error, the heading of the system is unobservable, which is also confirmed in the observability analysis conducted in [52]. Therefore, aiding sensors or measurements are needed to further correct the error of the system.

However, when integrating the indoor map information with the INS system using the APF, whose solutions are shown in Figure 6b, Figure 9b and Figure 12b, the estimated position accuracy is dramatically increased, as the map information can strongly constrain the heading of the system by deleting the ineffective particles. We can see from Figure 6b, Figure 9b and Figure 12b that, by performing the MA method, the end point and starting point of each test almost overlap, which can indirectly demonstrate that the proposed MA method is available. Moreover, compared to the MA-derived solution with the reference trajectory in different scenarios, the RMS (root mean error) of the estimation error and the mean of the estimation error could be controlled within 2 m. Furthermore, when compared with the other traditional particle filters, the APF with a cascade structure has a low computational burden because the update rate of the PF has been changed from an IMU data output rate (40 Hz) to a step-detection update rate.

Additionally, from the MM–MA derived positions in Figure 7a, Figure 10a and Figure 13a, we can see that, around the corner, the system does not perform the MM method. This outcome is because at the corner, there are at least two map line segments that satisfy the MM distance threshold. This is one drawback of MM method; therefore, to avoid wrong matching for this situation, no MM will be used. 

From Table 2, it can be seen that with the decrease in the number of particles, the RMS of the MM-only estimation error increases. This is because the accuracy of the PF associates with the number of particles, according to the law of large number. When the number of particles, N, is an infinite number, the value of the right part in Equation (6) becomes infinitely close to its left part, which means that the particles in PF can fully represent the PDF (probability density function) of the system. Usually, thousands of particles are needed to implement PF for real applications, and the more particles the system has, the more accurate the navigation solution will be. However, increasing the number of particles reduces the computational speed. Traditional particle filter-based indoor navigation methods need thousands of particles to implement indoor navigation. However, in this research, using the proposed method and algorithm structure, a lesser number of particles was needed.

By comparing the error of the MA-only method and MM–MA method, we can see that, for 1500 particles, the RMS error of the MA-only method is 1.45 m, which is smaller than the MM–MA method 1.56 m. By decreasing the particles to 1200, the difference between two methods is 0.002. The error rate of the MA-only method increases faster than the MM–MA method. When only 600 particles are used in the program, the RMS error of the MM–MA method is smaller than the MA-only method, as the number of the particles cannot be well represented in the system’s PDF. By continuing to reduce the number of particles to 500, the MM–MA method performs better than the MA-only method. 

## 5. Conclusions

In this research, a totally non-infrastructure based and low-cost indoor navigation method is proposed, which is inexpensive and time-saving compared with existing methods. Only indoor map information and smartphone built-in sensors are used in this algorithm. No pre-surveying, pre-installation, or additional aiding sensors are needed for the system. Therefore, the method makes the indoor navigation system more accessible, applicable and practical for users. 

Indoor map information and the MEMS sensors are integrated through a two-layer APF/KF structure algorithm. Mathematically, from the update frequency of the two filters, it could be shown that the two-layer filter structure decreases the computational burden of the system. By performing real-world experiments, we can obtain the following conclusions: the proposed method can achieve an ideal navigation calculating precision; and the MA-based APF method can dramatically improve the accuracy of the INS-derived navigation solution. Moreover, when the number of the particles is limited to decrease the computational burden of the system, the MM method can be applied to further optimize the MA-based APF results.

## Figures and Tables

**Table 1:** Sensors of Samsung S4 and Xiaomi 3.

Sensor	Samsung S4	Xiaomi 3
	Model	Model
**Accelerometer**	STM K3DH	MPU6050
**Gyroscope**	STM K3G	MPU6050

**Table 2:** Indoor positioning performance using different particles in test 3.

Particles	Algorithm	Error (m)			
		Max.	Min.	Mean	RMS
**1500**	ASIR(MA)	2.97	0.001	1.23	1.47
	MM-ARIR	3.15	0.0001	1.34	1.57
**1200**	ASIR(MA)	5.15	0.003	1.67	1.97
	MM-ARIR	5.35	0.003	1.69	1.98
**600**	ASIR(MA)	4.18	0.004	1.70	2.37
	MM-ASIR	4.18	0.004	1.78	2.32
**500**	ASIR(MA)	3.72	0.008	1.79	2.43
	MM-ARIR	3.74	0.008	1.66	2.22
